# Cross-Talk among Polymorphonuclear Neutrophils, Immune, and Non-Immune Cells via Released Cytokines, Granule Proteins, Microvesicles, and Neutrophil Extracellular Trap Formation: A Novel Concept of Biology and Pathobiology for Neutrophils

**DOI:** 10.3390/ijms22063119

**Published:** 2021-03-18

**Authors:** Chang-Youh Tsai, Song-Chou Hsieh, Chih-Wei Liu, Cheng-Shiun Lu, Cheng-Han Wu, Hsien-Tzung Liao, Ming-Han Chen, Ko-Jen Li, Chieh-Yu Shen, Yu-Min Kuo, Chia-Li Yu

**Affiliations:** 1 Division of Allergy, Immunology & Rheumatology, Taipei Veterans General Hospital, National Yang-Ming Chiao-Tung University, Taipei 11217, Taiwan; cwliu@vghtpe.gov.tw (C.-W.L.); darryliao@yahoo.com.tw (H.-T.L.); meikankimo@yahoo.com.tw (M.-H.C.); 2 Department of Internal Medicine, National Taiwan University Hospital, National Taiwan University College of Medicine, Taipei 10002, Taiwan; hsiehsc@ntu.edu.tw (S.-C.H.); b89401085@ntu.edu.tw (C.-S.L.); chenghanwu@ntu.edu.tw (C.-H.W.); dtmed170@yahoo.com.tw (K.-J.L.); tsichhl@gmail.com (C.-Y.S.); 543goole@gmail.com (Y.-M.K.); 3 Institute of Clinical Medicine, National Taiwan University College of Medicine, Taipei 10002, Taiwan

**Keywords:** polymorphonuclear neutrophil, neutrophil extracellular trap (NET), ectosome, exosome, low-density granulocyte, polymorphonuclear myeloid-derived suppressor cell (PMN-MDSC), antibody-dependent cellular cytotoxicity (ADCC), trogocytosis, systemic lupus erythematosus (SLE)

## Abstract

Polymorphonuclear neutrophils (PMNs) are traditionally regarded as professional phagocytic and acute inflammatory cells that engulf the microbial pathogens. However, accumulating data have suggested that PMNs are multi-potential cells exhibiting many important biological functions in addition to phagocytosis. These newly found novel activities of PMN include production of different kinds of cytokines/chemokines/growth factors, release of neutrophil extracellular traps (NET)/ectosomes/exosomes and trogocytosis (membrane exchange) with neighboring cells for modulating innate, and adaptive immune responses. Besides, PMNs exhibit potential heterogeneity and plasticity in involving antibody-dependent cellular cytotoxicity (ADCC), cancer immunity, autoimmunity, inflammatory rheumatic diseases, and cardiovascular diseases. Interestingly, PMNs may also play a role in ameliorating inflammatory reaction and wound healing by a subset of PMN myeloid-derived suppressor cells (PMN-MDSC). Furthermore, PMNs can interact with other non-immune cells including platelets, epithelial and endothelial cells to link hemostasis, mucosal inflammation, and atherogenesis. The release of low-density granulocytes (LDG) from bone marrow initiates systemic autoimmune reaction in systemic lupus erythematosus (SLE). In clinical application, identification of certain PMN phenotypes may become prognostic factors for severe traumatic patients. In the present review, we will discuss these newly discovered biological and pathobiological functions of the PMNs.

## 1. Introduction

Polymorphonuclear neutrophils (PMNs) have long been known as an essential element of the innate immune system, but have been ignored of their participation in the adaptive immune responses or autoimmune reaction. PMNs usually arrive first to the sites of infection for carrying out phagocytosis of the microbial pathogens in association with release of proteinases and oxidants to kill the invaders. During the process, an acute inflammation concomitant with damage to the neighboring tissues occurs. PMNs are also blamed to be the culprits of certain non-infectious inflammatory diseases such as inflammatory bowel disease, acute gouty arthritis, and reperfusion injury after a cardiovascular attack or organ transplantation. However, in a series of in vivo studies, Sendo et al. [1,2,3] found that depleting PMNs by anti-PMN antibodies in rats led to the abolishment of a number of immune effector functions such as delayed-type hypersensitivity [1], tumor inhibitory activity [2], and antibody-producing capacity [3]. These results may suggest that PMN is not merely a terminally differentiated phagocytic cell against bacterial pathogens and an elicitor of acute inflammation, but can actively participate in the immune responses. More recently, Brinkmann et al. [4] disclosed that PMNs, upon activation, extruded their nuclear DNA and histones in association with the attached granule proteins to form the so-called “neutrophil extracellular traps (NET)” to trap the environmental foreign invaders. Furthermore, neutrophils are found to consist of heterogeneous populations, not only including inflammatory but also of angiogenic subsets, as proposed by Christofferson et al. [5]. These authors showed that the pro-inflammatory PMNs (CD116^+^ Gr-1^+^ CXCR4^hi^) were recruited to the injured sites by CXC chemokines ligand 2 (CXCL2). On the other hand, the pro-angiogenic PMNs (MMP9^hi^CXCR4^hi^) were attracted in response to vascular endothelial growth factor A (VEGF-A). Denny et al. [6] identified a high-density (HDG) and a low-density granulocytes (LDG) in the blood circulation of patients with systemic lupus erythematosus (SLE). More interestingly, the LDGs exhibit a striking tendency to NET formation, suggesting this subset is one of the proinflammatory subtypes [7]. Deniset et al. [8] further identified an Ly6G^hi^ (mature) and an Ly6G^int^ (immature) subsets of PMNs in the spleen with their main function being eradicating *S. pneumoniae* [9]. Accordingly, PMNs are currently considered as complex but enigmatic cells in conducting novel biological functions, including effectors of the innate immune responses, tumor killing, autoimmunity, chronic inflammatory reaction, anti-inflammation, and wound repair [10]. In this mini-review, we will discuss in detail the novel biological activities of PMNs in health and disease states consecutively.

## 2. The Newly Found Biological Functions of PMNs

A number of novel biological functions of PMNs have been successively found, which render them an important member of the immune network and crucial regulator involved in biology and pathobiology.

### 2.1. Production of Cytokines, Chemokines, and Growth Factors by the PMN for Immune Modulation

Several investigators confirmed that PMNs could produce TNF-α [11,12,13], IL-1β [12,14,15], IL-8 [16,17,18,19], TGF-β1 [20,21], IL-1ra [15,22,23,24], IL-6 [12,13,25], IFN-α [12], G-CSF [12], M-CSF [12], GM-CSF [26], and IL-12 [27]. Critical reviews of these neglected roles of PMNs in the afferent limb of the immune response have been published by Lloyd et al. [12] and later by Cassatella MA [28]. Notably, IL-12 produced by PMNs can facilitate T cell release of IFN-γ [29], which then positively sends feedback to further stimulate the production of IL-12 by themselves as a potent PMN activator [30]. Besides, PMN can express MHC class II antigens following exposure to IL-3, IFN-γ, and GM-CSF, resembling an antigen presenting cell (APC) [31,32]. These intimate interactions between PMNs and T lymphocytes undoubtedly can mediate various physiological as well as pathophysiological processes, including immune responses, inflammation modulation, fetal-maternal tolerance, tumor immunity, autoimmune diseases, inflammatory diseases, and cardiovascular diseases [33].

We have long been devoted in the investigations of cytokine/chemokine gene expression in PMNs of patients with SLE. Hsieh et al. [34] first explored the decreased spontaneous and lipopolysaccharide (LPS)-stimulated IL-8 production by SLE-PMN. In addition, SLE-PMN was found to be hypo-responsive to IL-8 stimulation because of a defective expression of IL-8 chemotactic receptor, CXCR2, on its surface [35]. Later, Hsieh et al. [36] found a defective spontaneous and LPS stimulated IL-1 receptor antagonist (IL-1ra) gene expression in SLE-PMN. For elucidating the effects of abnormal SLE-PMN cytokine expression on autologous T cell response, Yu et al. [37] unveiled that the aberrant peritoneal PMN cytokine expression could skew the autologous mononuclear T cells toward Th2 immune response in autoimmune MRL-*lpr*/*lpr* mice.

**Figure 1:**
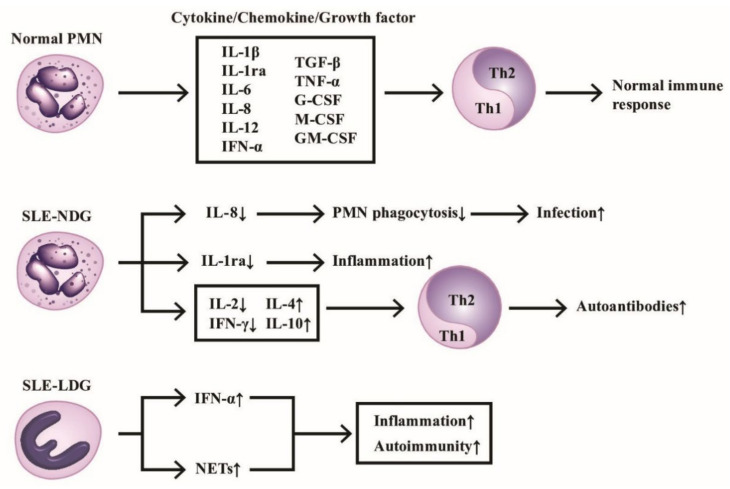
The production of cytokines/chemokines/growth factors by PMNs from normal, normal density (SLE-NDG), and low density (SLE-LDG) granulocytes of patients with SLE. The effects of these mediators on Th1/Th2 differentiation and pathological roles are summarized. IL-1ra: IL-1 receptor antagonist, NETs: neutrophil extracellular traps, CSF: colony-stimulating factor. Th: helper T; GM-CSF: granulocyte-macrophage colony stimulating factor; IFN: interferon; IL: interleukin; TGF; transforming growth factor; TNF: tumor necrosis factor. ↑ denotes increase, ↓ denotes decrease.

The production of cytokines/chemokines/growth factors by PMNs of normal individuals and SLE patients in modulating immunological and inflammatory responses are shown in Figure 1, which highlights the homeostasis of Th1/Th2 immune response maintained by normal PMN via releasing balanced cytokines/chemokines/growth factors. In contrast, the deranged cytokine production by either normal or low-density neutrophils from SLE patients skews Th response toward Th2 and subsequently enhances inflammatory and autoimmune outcomes.

### 2.2. The Physiological and Pathophysiological Roles of the Exocytosed Molecules from PMNs

PMNs are found not only to produce immune-related mediators implicated in the immune network as discussed in the above subsection but to release the tangled webs called neutrophil extracellular traps (NETs) on activation. Besides, PMNs can excrete granule proteins and microparticles, including ectosomes, exosomes, apoptotic bodies, and other microvesicles to affect the physiological or pathophysiological functions of the remote cells/tissues. We are going to discuss first in detail the physiologic formation of NETs and then, the pathological or dark side of diminished NET clearance in eliciting autoimmune diseases and vasculitis.

#### 2.2.1. The Physiology and Molecular Basis of NET Formation

Neutrophils can engulf and kill the invaded pathogens mainly by antimicrobial granules after fusing with them. However, Brainkmann et al. [4] unexpectedly found that the activated PMN could release granule proteins, which are attached with the extruded nuclear chromatin for trapping and killing the extra-corpuscular microbial pathogens. The authors observed that when PMNs are stimulated by bacterial endotoxin or proinflammatory cytokines, they can extrude a web-like structure containing DNA, histones, and different granule proteins such proteinase 3 (Pr3), myeloperoxidase (MPO), elastase (Ela), LP-33, and other granule enzymes, which can then effectively catch and destroy the trapped microbes.

It has been demonstrated that microbes *per se* and their products, biochemical stimuli, high concentration of calcium ion, immune complexes, platelets and damage-associated molecular patterns (DAMPs) can trigger NET formation or “NETosis”. The molecular basis of NET formation can be arbitrarily divided into NADPH-oxidase (Nox)-dependent and Nox-independent pathways. In addition, certain internal factors (ROS production and/or transcription factor activation) and external factors (alkaline pH and hypertonic conditions) may also affect individual NET pathways [38,39,40].

Nox-dependent NETosis (PMA, LPS or bacteria activation) can induce Nox-ROS whereas Nox-independent NETosis (A23187 or ionomycin) induces mitochondria-ROS, Ca^2+^ and peptidylarginine deimidase 4 (PAD4) [40]. Both Nox-ROS and mitochondria-ROS can activate different sets of kinases specific for Nox-dependent (ERK1/ERK2) and Nox-independent (ERK1/2, JNK, FAK-2, IKK), as well as common kinases (Akt, *p*38, Src) [41,42,43,44], leading to transcriptional firing [39]. The Nox-independent and NET pathway-induced high [Ca^2+^] influx can activate PAD4 and cause histone hypercitrullination [45].

It is believed that Rac proteins (Rac 1, Rac 2, and Rac 3) play an important role in regulating PMN responses to inflammatory signals. These responses include actin remodeling, chemotaxis, and superoxide production by NADPH oxidase [46]. Gavillet et al. [47] reported that Vav-Rac-Pak signaling axis is involved in NET formation. Tatsiy et al. [48] further lineated that TAK1, *p*38 MAPK, or MEK signaling pathway acts on the early events while Syk or PI3K pathway acts on the late events in PAD4-dependent, ROS-independent NETosis. Boeltz et al. [49] demonstrated that nuclear envelope rupture can be the prerequisite as well as hallmark of NET formation. Neubert et al. [50] noted that rupture of nuclear envelope in NETosis appeared to be a distinct process from the ordinary nuclear envelope breakdown or dissolution. Galdberg et al. [51] further confirmed that B-type lamins (B1 and B2) formed thinner but highly organized meshwork than A-type lamins (A, C), which are crucial for the integrity and elasticity of the nuclear envelope. Based on these findings and further studies, Li et al. [52] concluded that nuclear translocation of PKCα acts as the kinase and induces lamin B phosphorylation to disassemble nuclear membrane in the process of NETosis.

#### 2.2.2. The Dark Side of NET: Its Decreased Clearance Induces Autoantibody Production in Autoimmune Diseases

Several types of NETosis have been reclassified in recent years, such as vital [53], fatal [54,55], and a variety of suicidal NETosis based on the presence of mitochondrial DNA [38]. Accordingly, aberrant NET formation may play a pathological role in inducing immunological/inflammatory disorders, including SLE [56,57], RA [58], multiple sclerosis [59], adult-onset Still’s disease [60], inflammatory bowel diseases [61], anti-cytoplasmic antibody (ANCA)-associated vasculitis (AAV) [62], and diabetes (type 1 and type 2) [63]. Undoubtedly, NET structures can serve as scaffolds for thrombus formation [64] or provide autoantigens in inducing autoantibodies production in the condition of defective NET clearance. The clearance of NETs are found dependent on complement component C1q [65], DNase 1 [65], and C-reactive protein [66].

**Figure 2:**
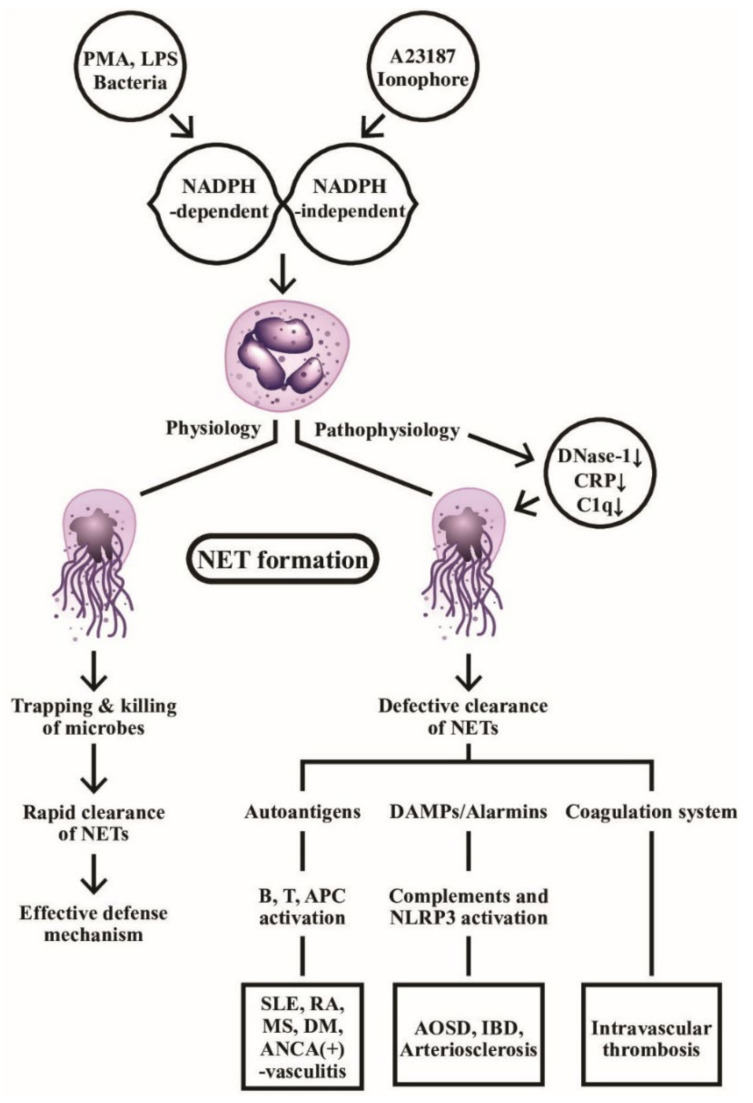
The physiological and pathophysiological roles of NADPH-dependent and NADPH-independent neutrophil extracellular traps (NETs) formation are demonstrated. Left: the web-like NETs with attached granule enzymes not only effectively trap but kill the invasive microbial pathogens as an important defense mechanism in normal physiological condition. Right: Defective NET clearance due to defective DNase-1, CRP, and C1q functions may lead to longstanding presence of the NET in the blood and tissues. These molecules may become autoantigens, damage-associated molecular patterns (DAMP)/alarmins stimulants, or coagulation system activator to induce autoimmunity, inflammatory reaction, and intravascular thrombosis. PMA: phorbol myristate acetate; LPS: lipopolysaccharide; AOSD: Adult onset Still’s disease; MS: multiple sclerosis; IBD: inflammatory bowel disease; DM: diabetes mellius; NLRP: Nucleotide-binding oligomerization domain, Leucine rich Repeat and Pyrin domain containing; APC: antigen presenting cell; ANCA: anti-neutrophil cytoplasmic antibody; SLE: systemic lupus erythematosus; RA: rheumatoid arthritis; CRP: C-reactive protein; NADPH: nicotinamide adenine dinucleotide phosphate-hydrogen ion.

Obviously, the persistent presence of NETs can break down the self-tolerance by providing autoantigen sources to stimulate autoantibody production in the immune system, such as anti-citrullinated protein antibodies in RA [67], anti-dsDNA and anti-histone antibodies in SLE [68], as well as anti-MPO or andanti-Pr3 autoantibodies in AAVs [69]. Moreover, NET components can accelerate inflammatory reactions via the activation of complement and coagulation systems [70,71], or serving as DAMP to activate NRLP3 inflammasome [60,72]. NETs can also stimulate B cells [73], APCs [74], and T cells [75] to propel autoimmune reactions. It is quite intriguing that NETs can link to resolution as well as induction of inflammation [76,77]. Thereby, NET formation provides a connection among infection, autoimmunity, inflammation, endothelial dysfunction, intravascular thrombosis/atherogenesis and anti-inflammation, as shown in Figure 2. It should be emphasized that the effective clearance of NET formation is critical for physiological defense function against microbial invasion. On the contrary, defective NET clearance can become a pathological process in causing autoimmunity, inflammation and intravascular coagulopathy.

### 2.3. Extrusion of Granule Proteins, Ectosomes, and Exosomes from PMNs for Remote Cell-Cell Communications

In addition to the roles of NETs in physiology and pathophysisology, the exocytosis of granule proteins, ectosomes, and exosomes from PMNs can mediate the immune modulation and remote cell-cell communication between PMNs and other immune and non-immune cells. We will go on with these issues in detail in the following subsections.

#### 2.3.1. The Effects of Released Granule Proteins in Modulating Innate and Adaptive Immune Responses

In general, neutrophil granules can be divided into four groups according to the different maturation stages: (1) primary or azurophil granules; (2) secondary or specific granules; (3) tertiary granules including arginase and gelatinase, and (4) secretory vesicles such as ectosomes and exosomes [78]. The molecules categorized as the azurophil granule proteins include MPO, Pr3, bactericidal permeability-increasing protein (BPI), defensins, azurocidin (CAP37), elastase and cathepsins. The molecules categorized as the secondary granule proteins are lactoferrin (LF, a vitamin B12-binding protein), lysozyme, and properdin [79].

The impact of these granule proteins on immune responses is largely implicated in APC-T cell immunity. MPO can modulate immune responses by either CD4^+^T cell activation [80] or dendritic cell suppression [81]. Alpha-defensins and human neutrophil peptides (HNP1) can induce NF-κB signaling in CD4^+^T cell [82] and the production of IFN-α by plasmatoid dendritic cells (*p*DC) [83]. Elastase (Ease) can potently promote Th17 response [84] but simultaneously induces DC production of TGF-β for suppressing T cell proliferation [85]. LL-37 (cathelicidin, derived from CAP18) is a chemoattractant for CD4^+^T cells [86] but can inhibit Toll-like receptor (TLR) signaling in DC [87]. Chertov et al. [88] found that cathepsin G exhibited more potent chemo-attractive activity for monocytes than azurocidin. The same authors also found that azorocidin/CAP37 was a T-cell chemoattractant protein [89]. Heizelmann et al. [90] discovered that azurocidin and fucoidin could modulate LPS-induced monocyte activation. Schwaeble et al. [91] reported that the secondary granule protein, properdin, can indirectly enhance PMN, macrophage, and mast cell migration. The tertiary granule protein, arginase, can suppress T cell proliferation [92] and gelatinase can induce DC migration as well as T cell priming in DTH [93].

Li et al. [94] discovered that LFs could be expressed on the surface of PMN. After contact with CD4^+^T cells, the LFs were transferred from PMNs to CD4^+^ cells, suppressing IFN-γ but enhancing IL-10 expression in CD4^+^T cells. Legrand et al. [95] further found LF could interact with proteoglycans and different receptors on the surface of innate (NK cells, PMNs, macrophages, basophils/mast cells) and adaptive (lymphocytes and APCs) immune cells. Through these interactions, LF can modulate the migration, differentiation, and functions of immune-related cells. In pathobiological sense, decreased LF expression in SLE-PMN can skew CD4^+^T cells toward Th2 immune responses in SLE patients [94]. In addition, LL-37 was found able to form complexes with DNA. The LL-37-DNA/anti-DNA complexes are easily recognized by FcγRII on *p*DCs. The endocytosis of the complexes with its recognition via TLR9 in *p*DC leads to type I IFN production and subsequent immune activation [96].

**Figure 3:**
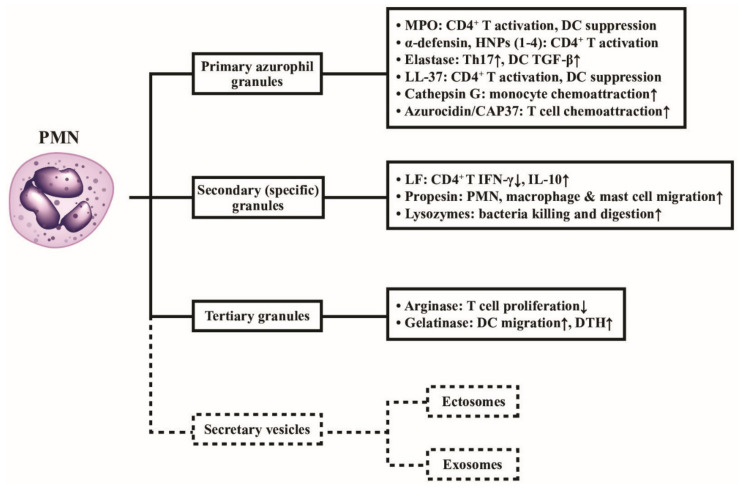
Classification of neutrophil granules and their immunological functions. Different granule proteins included in the primary, secondary, and tertiary granules are summarized in brackets. The vesicles extruded from PMNs are simply divided into ectosomes and exosomes. The formation, composition, and biological/pathobiological functions of the secretary vesicles are not shown in the figure and are instead described in detail in Figure 4. MPO: myeloperoxidase; HNP: human neutrophil peptide; IL: interleukin; IFN: interferon; DC: dendritic cell; DTH: delayed-type hypersensitivity; LF: lactoferrin; CD: cluster of differentiation; CAP: cationic antimicrobial protein; LL-37: cathelicidin.

**Figure 4:**
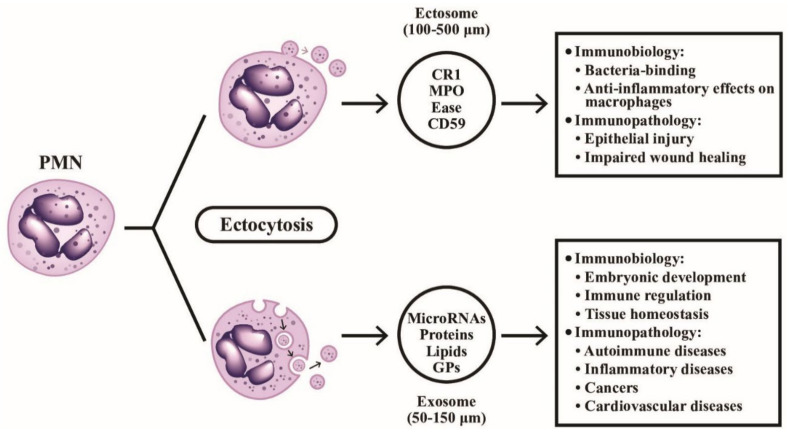
The formation, particle size, contents, and detailed immunobiological/immunopathological functions of ectosomes and exosomes extruded from PMN are demonstrated. The ectosomes are microvesicles containing granule proteins and surface membrane-expressed components such as complement receptors and CD59. The ectosomes directly detach from cell surface without deepening into cytoplasm. On the contrary, exosomes vaginate inward into the cytoplasm and take up cytoplasmic DNAs, RNAs, glycoproteins and lipids before extrusion from the cells. CR1: complement receptor type 1; MPO: myeloperoxidase; Ease: elastase; GP: glycoprotein; CD: cluster of differentiation.

The effects of different neutrophil granule proteins on immune responses are shown in Figure 3, outlining the functions of the different classes of released granule proteins in regulating immune responses in addition to digesting the engulfed pathogens. Besides, the secretary microvesicles from PMNs can circulate and influence the functions of remote cells by granule proteins and nucleic acids, as depicted in Figure 4.

#### 2.3.2. The Extracellular Microvesicles Budding or Extruded from PMNs for Remote Cell-Cell Communications

Cocucci et al. [97] disclosed that human PMNs could release membrane microvesicles ranging from 5 to 200 nm in diameter, in response to specific activator, through the mechanism of exocytosis. These “exocytosed” microvescicles enclosed by a lipid bilayer can be divided into ectosomes and exosomes. The formation of these vesicles depends on local micro-domains assembled in the endocytic membranes for exosomes and in the surface membrane for ectosomes. The micro-domains are composed of proteins and different types of RNA associated with cytosolic surface. The membrane invaginates inwards to form the exosome precursors (50–150 µm) and buds outwards to form ectosomes (100–500 µm). After exocytosis, these two kinds of microvesicles navigate via extracellular fluid to the remote tissues for cell–cell communications. After fusion with plasma membrane of the target cells via endocytosis, the changes in the biology and physiology of the target cells can be achieved by the two microvesicles [98].

#### 2.3.3. Specific Functions of Ectosomes Extruded from PMNs

Hess et al. [99] showed that ectosomes released from human PMN express clusters of type 1 complement receptor and are co-localized with MPO, elastase and CD59 to bind efficiently to the opsonized bacteria at the site of inflammation in vitro. On the contrary, Gasser et al. [100] found that PMN-derived ectosomes not only fail to exhibit proinflammatory effect on the human macrophages to facilitate their release of IL-8 or TNF-α, but conversely stimulate their TGF-β release. In addition, the ectosome-cell contact is sufficient to block the phagocytosis of macrophages triggered by cytochalasin D inhibition. These results have suggested that PMN-derived ectosomes (PMN-Ect) can exert potent anti-inflammatory activities in the early stage of inflammation. Eken et al. [101] also confirmed that PMN-Ect could interfere with zymosan A (Zym A) activation of macrophages via inhibition of NF-κB *p*65 phosphorylation and NF-κB translocation. The Mer receptor tyrosine kinase (Mer TK) and PI_3_K/Akt pathways are presumed to play a key role in this immunomodulatory effect of PMN-Ect. The same authors later confirmed that PMN-Ect could activate another two signaling pathways, an immediate Ca^2+^ influx and a rapid release of TGF-β, which are independent of Mer TK signaling [102].

Pathophysiologically, the deposition of PMN-Ect-derived MPO onto the inflamed intestinal epithelial cells (IECs) during neutrophil trans-epithelial migration (TEM) would cause loss of epithelial cadherin and thus enhance epithelial injury. This epithelial injury further facilitates neutrophil recruitment via high level of active MMP-9 to cleavage the desmoglein-2 (DSG-2). The enzymatic activity of MMP-9 can further disrupt the intercellular adhesion of IEC to promote TEM of PMN [103]. Slater et al. [104] also demonstrated that PMN-Ect-derived MPO can further retard the ongoing wound healing in IEC by impairing their migration and proliferation through the inhibition on actin dynamics and cell immigration, and the cell cycle arrest [104].

##### Immunobiology and Immunopathology of Exosomes Extruded from PMN in Health and Disease

Exosomes are exocytotic membrane-derived microvesicles containing nucleic acids, proteins, lipids, amino acids, glycoconjugates, and metabolites in biological fluids for mediating remote cell-cell communication mainly via delivery of proteins and non-coding RNAs (ncRNAs) [105]. The cargo of exosomes allows these bioactive molecules to play roles in many physiological functions such as embryonic development, immune regulation and tissue homeostasis, and in pathological states. These pathological conditions can be categorized into autoimmune connective tissue diseases, as reviewed by Zhu et al. [106], rheumatic inflammatory diseases as reviewed by Console et al. [107], and non-communicable diseases such as cancers or cardiovascular diseases as reviewed by Jimenez-Avalos et al. [108].

The immunological and immunopathological roles of ectosomes and exosomes released from PMN are illustrated in Figure 4. As show in this figure, it is worthy to know that the exocytosed microparticles from PMNs contain a broad spectrum of effector molecules (granule proteins in ectosomes, and proteins, DNAs, miRNAs, non-coding RNAs, and lipids in exosomes) mediating both immunological and immunopathological functions in embryonic development, immune regulation, autoimmunity, inflammation, tumor immunity, and cardiovascular diseases.

### 2.4. Cross-Talk among PMN and Other Immune-Related Cells via Trogocytosis

**Figure 5:**
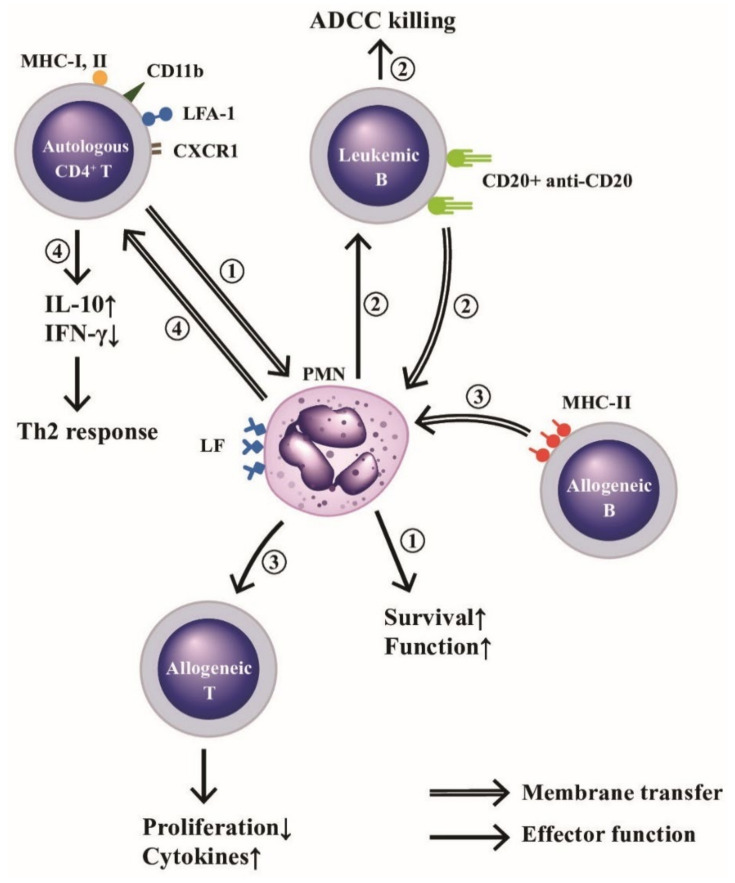
Membrane transfer among neutrophils and autologous CD4^+^T, allogeneic B or leukemic B cells for mediating respective effector function. (**1**) PMNs “trogocytose (bite up)” immunological synapse-associated molecules (HLA-I and II, CD11b, LFA-1, and CXCR1) to enhance their biological functions and survival rate. (**2**) PMNs trogocytose CD20-anti-CD20 complex from leukemia B cells and then kills these tumor cells by ADCC mechanism. (**3**) PMNs trogocytose MHC-II antigens from allogeneic B lymphocytes and then present MHC-II to allogeneic T cells to enhance proliferation and cytokine production of the allogeneic T cells, and (**4**) Autologous CD4^+^T cells trogocytose lactoferrins (LFs) expressed on PMNs to enhance IL-10 and IFN-γ production for skewing cells to Th2 immune responses. ADCC: antibody-dependent cell-mediated cytotoxicity. MHC: major histocompatibility complex; LFA: lymphocyte function-associated antigen; CD: cluster of differentiation; CXCR: cysteine-amino acid X-cysteine chemokine receptor; IFN: interferon; IL: interleukin; HLA: human leukocyte antigen.

One of the intercellular communication ways among immune-related cells relies on the immunological synapse formation with subsequent membrane exchange [109]. Whale et al. [110] demonstrated that bovine PMN passively acquired membrane lipids and integral membrane from apoptotic and necrotic cells. The same authors further confirmed that MHC class II antigens were also passively transferred from allogeneic B cells to PMNs to enhance the proliferative response and cytokine gene expression of alloreactive T cell lines [111]. In addition, Li et al. [112] proved that an active membrane transfer from CD4^+^T cells to autologous PMNs after contact via the immunological synapse containing HLA class-I and II, CD11b, leukocyte function antigen-1 (LFA-1), and CXCR1 would transduce signals against extrinsic apoptotic and MAP kinase activities to enhance neutrophil functions. Recently, Valgardsdottir et al. [113] demonstrated that human PMNs could conduct trogocytosis rather than phagocytosis of anti-CD20 antibody-opsonized B cells in patients with chronic lymphocytic leukemia. Clinically significantly, Li et al. [112] further demonstrated that decreased total trogocytosis from PMNs to mononuclear cells (MNCs) suppressed IL-2 production by MNCs in the patients with SLE. A critical review of defective SLE-PMN functions in the immune responses has been published by Tsai et al. [114]. The effects and outcomes of the trogocytosis among PMNs, autologous CD4^+^T, allogeneic CD4^+^T or allogeneic B, are illustrated in Figure 5, which indicates the membrane transfer between PMNs and other immune-related cells or antibody-targeted tumor cells after cell-cell contact to modulate the immune responses, granule protein-mediated cell survival, and tumor immunity including antibody-dependent cellular cytotoxicity (ADCC) exerted by neutrophils.

### 2.5. The Biological Significance of Cross-Talk between PMNs and Nonimmune-Related Cells

It has been confirmed that PMNs act not only as initiators in the early stage but also as firefighters in the late stage of inflammation. Following the same line, PMNs may probably play homeostatic roles in infectious and non-infectious inflammation via interactions with nonimmune-related or somatic cells. The cross-talk of PMNs with non-immune related cells such as epithelial cells, periodontal tissue cells, or microbes are discussed in the following subsections.

#### 2.5.1. Neutrophil–Epithelial Interactions

When PMNs migrate to the sites of inflammation in mucosa-lined tissues such as lungs and gastrointestinal tracts, these PMNs have to “talk” to mucosal epithelial cells to “abolish” the damage they exert to the surrounding host tissues. Dysregulated or excessive PMN transmigration would lead to bystander-induced tissue damage as observed in inflammatory mucosal diseases. It is now well realized that adhesion-based PMN-epithelial interactions involve specific cell adhesion molecules such as β2 integrins, CD11b/18 on neutrophils [115]. Similarly, the active inflammatory processes in tissues lined with columnar epithelial cells are characterized by abundant PMN migration that would induce pathological PMN-epithelial interactions and elicit mucosal inflammatory diseases [116,117].

#### 2.5.2. The Cross-Talk among Microbes, Neutrophils, and Periodontal Tissue in the Induction of Periodontitis and Inflammatory Bone Loss in Oral Cavity

Periodontitis is a chronic inflammatory disease that destroys both gingival soft tissues and bone. PMNs are required for the important immunomodulatory functions in periodontium because the absence of PMNs may facilitate overproduction of IL-17 to drive inflammatory bone loss [118]. On the contrary, Chakravarti et al. [119] found that PMN could directly induce osteoclastic bone resorption by stimulating membrane-bound receptor activator of NF-kB ligand (RANKL) expression. Abe et al. [120] disclosed that PMN could secrete B lymphocyte stimulator (BLyS) and APRIL to enhance the survival of RANKL-expressing B and plasma cells to ease bone loss happening in periodontitis. Hajishenallis et al. [118] also discovered that periodontitis is a dysbiotic disease in which PMN becomes the target of immune subversion by periodontal bacteria via microbial exploitation of C5a receptor-1 and Toll-like receptor-2 on PMN. The scenario may indicate the microbe-neutrophil-periodontium interactions in the induction of periodontitis and the neighboring bone loss.

**Figure 6:**
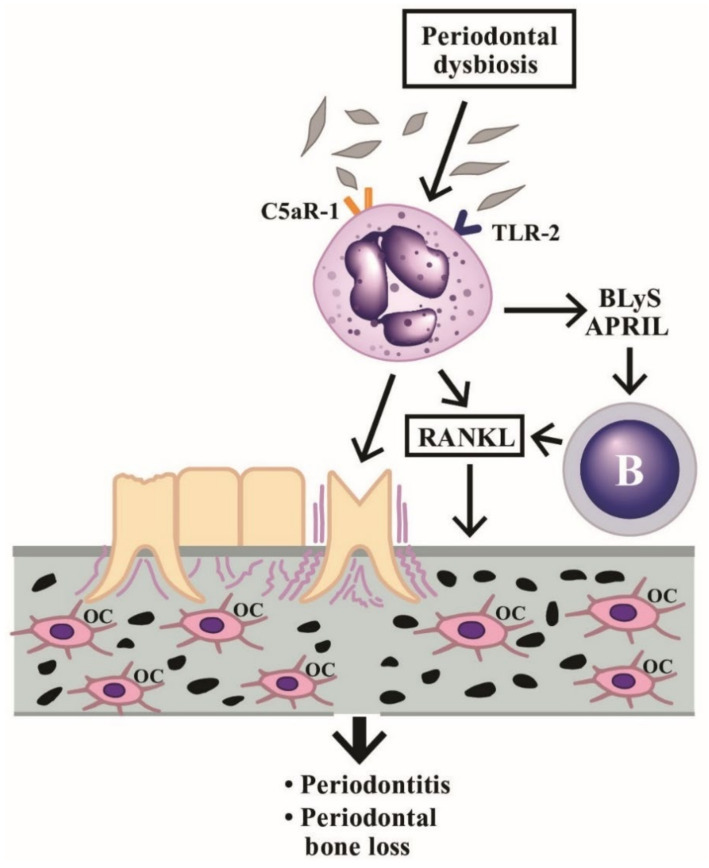
Induction of periodontitis and periodontal bone loss by PMNs initiated by periodontal dysbiosis. The periodontal dysbiotic bacteria bind to C5aR-1 and TLR-2 on PMNs. The binding stimulates BLyS and APRIL release from PMNs, which then activate B cells to produce RANKL. The RANkL binds to RANK receptors on macrophages to facilitate these cells differentiating to osteoclasts (OC). Finally, periodontitis and bone loss in the periodontal tissue occur. BLyS: B Lymphocyte Stimulator; APRIL: A PRoliferation-Inducing Ligand; RANK: Receptor Activator of Nuclear factor Kappa; RANKL: RANK ligand; TLR: Toll Like Receptor; C5aR: Complement fragment 5a receptor.

An illustration showing the cross-talk among dysbiosis, neutrophils, B cells, periodontal tissues, and bone tissue in inducing periodontitis and inflammatory bone loss is given in Figure 6, in which the implication of PMN’s novel functions in periodontal dysbiosis are emphasized. The stimulated PMNs not only induce periodontitis but increase production of B cell stimulators (BLyS and APRIL) to facilitate the B cell release of RANKL and enhance osteoclast activity for periodontal bone destruction.

## 3. Diverse Effector Functions Mediated by Neutrophil Phenotypes with Plasticity in Health and Disease

Recent investigations unveiled that diverse neutrophil phenotypes with plasticity play roles in different unique functions in addition to the action against infections. This neutrophil diversity may emerge in situations of (1) pregnancy, (2) systemic autoimmunity, and (3) tumor immunity. We will discuss the particular effector functions of these heterogeneous neutrophil subpopulations in the following subsections.

### 3.1. Neutrophil Diversity in Pregnancy

**Figure 7:**
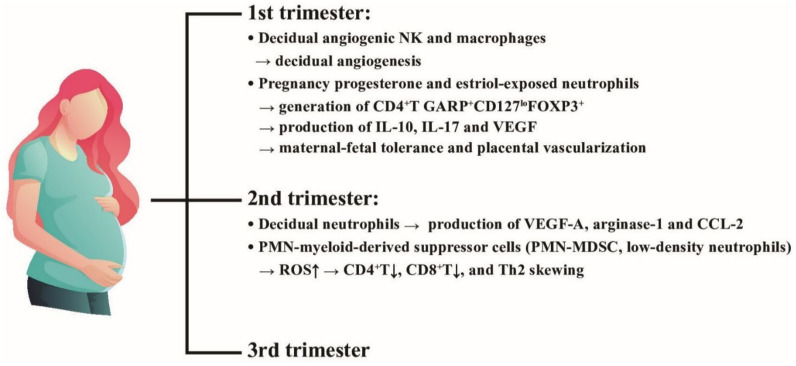
The effects of placental decidual neutrophils and neutrophil myeloid-derived suppressor cells (PMN-MDSC) on the placental angiogenesis and maternal-fetal tolerance in the first and second trimesters. NK: natural killer cells, ROS: reactive oxygen species, VEGF: vascular endothelial growth factor; CD: cluster of differentiation; IL: interleukin; FOXP3: Forkhead box P3; GARP: Glycoprotein-A Repetitions Predominant gene; CCL: cysteine-cysteine chemokine ligand.

Tabiasco et al. [121] demonstrated that the decidual leukocytes in the first trimester are composed of uterine natural killer cells and macrophages specialized for early placental decidual angiogenesis. Furthermore, Amsalem et al. [122] found that placenta decidual PMNs expressed high level of neutrophil activating markers and angiogenesis-related proteins, VEGF-A, arginase-1 and CCL2, in the second trimester. Interestingly, an immunosuppressive neutrophil population, low density neutrophils (LDNs) or polymorphonuclear myeloid-derived suppressor cells (PMN-MDSCs), were found in the blood and placenta of pregnant women for suppressing CD4^+^ and CD8^+^T cell proliferation via ROS production [123,124]. This particular PMN subpopulation can skew helper T (Th) immune responses toward Th2 pathway [124]. Nadkarni et al. [125] reported that PMNs exposed to the pregnancy hormones, progesterone and estriol, could induce CD4^+^T cells to display GARP^+^CD127^lo^FOXP3^+^ phenotype by antigen activation. These particular neutrophil-induced regulatory T cells could produce IL-10, IL-17 and VEGF, and then establish the maternal-fetal tolerance as well as normal placental vascularization. In contrast, through a long-term observation, Christoforaki et al. [126] revealed that neutrophil to lymphocyte ratio (NLR) of more than 5.8 was exclusively found in early miscarriage during the first trimester, compared to those without fetal loss in the same period. The roles of neutrophil heterogeneity in inducing maternal-fetal tolerance and placental vascularization are demonstrated in Figure 7. It is quite intriguing that PMN can exert a novel biological function in maternal-fetal tolerance in the early (first trimester) and middle (second trimester) stages of pregnancy. Three different PMN subsets including pregnancy hormone-exposed PMNs, decidual PMNs, and PMN-myeloid-derived suppressor cells (PMN-MDSC) actively participate in maintaining pregnancy via immunoregulation until the third trimester.

### 3.2. A Distinct Class of Neutrophils, Low Density Granulocytes (LDG), in Systemic Autoimmunity

**Figure 8:**
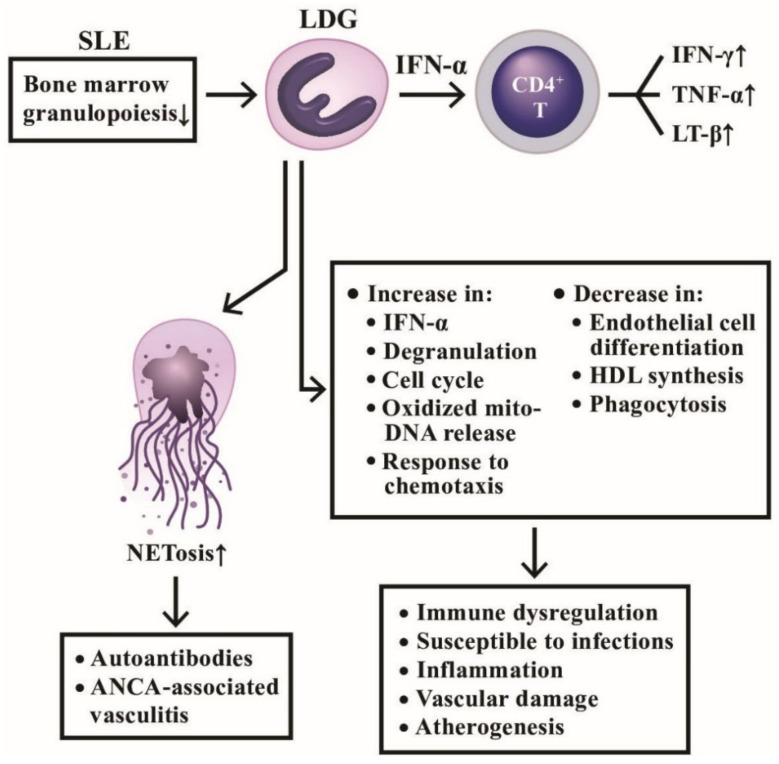
The productive and deleterious effects of low-density granulocytes (LDG) in patients with SLE. LDGs show a tendency to form NETs and induce a number of antinuclear antibodies and anti-neutrophil cytoplasmic antibodies (ANCAs) to mediate autoimmune diseases as shown on the left side. In addition, LDGs can secrete large amount of IFN-α to facilitate CD4^+^T cell’s production of cytokines and to exert a lot of immunological, inflammation, and vascular effects in patients with SLE as shown on right side. IFN; interferon; LT: lymphotoxin; TNF: tumor necrosis factor; HDL: high density lipoprotein; mito-: mitochondrial.

Hacbarth et al. [127] were the first to discover a subpopulation of “low buoyant density granulocytes” from adult SLE patients. Then, Nakou et al. [128] confirmed that these LDGs appeared in the peripheral blood and were correlated to the prevalence of skin diseases and vasculitis in lupus. Denny et al. [6] further disclosed that LDGs secreted increased amount of type I IFNs, TNF-α, and IFN-γ, but showed impaired phagocytic activity. These abnormal granulocytes could also exert endothelial cytotoxicity by interrupting the differentiation of endothelial progenitors to mature endothelial cells. To identify the nature of LDGs, Rahman et al. [129] reported that LDGs exhibited interferon 21-gene signature, various activation markers and lectin-like oxidized low-density lipoprotein receptor-1. These cells can activate CD4^+^T cells to produce proinflammatory cytokines, IFN-γ, TNF-α, and lymphotoxin-β, quite different from PMN-MDSC. Gene signature study of LDGs by Carlucci et al. [130] found that the most up-regulated genes in lupus LDGs are associated with vascular inflammation and non-calcified plaque burden (NCB). The SLE-LDGs were also found to disrupt high-density lipoprotein (HDL) functions and thereby could promote atherogenesis [130]. Kegerreis et al. [131] surveyed 92-gene module of SLE-LDGs and found that LDG gene signature was enriched in genes linked to neutrophil degranulation and cell cycle regulation induced by type I IFN and TNF. The authors concluded that LDG enrichment in SLE is reflecting increased granulopoiesis in the bone marrow, rather than peripheral neutrophil activation in the disease. Recently, Mistry et al. [132] used the transcriptomics and epigenetics to analyze SLE-LDGs and found 2 subpopulations of intermediate-mature and immature neutrophils with difference in NET formation, oxidized mitochondrial DNA release, chemotaxis, phagocytosis, degranulation, endothelial damage, and response to type I IFN stimulation. The pathological roles of LDG involved in lupus pathogenesis in schemed in Figure 8, disclosing that the pathogenic LDGs originated from immature bone marrow granulopoiesis in SLE patients display characteristic abnormal neutrophil functions with increased NETosis and mediate autoimmune/inflammatory reactions, vascular damage, as well as atherogenesis.

### 3.3. Roles of PMN-MDSC and Their Released Exosomes in Cancer Immunity and Autoimmune/Inflammatory Diseases

Recently, neutrophils attracted much attention in the context of cancer. PMNs can exert both anti- and pro-tumorigenic functions in the field of tumor immunology. The anti-tumor immunity can be achieved by antibody-dependent granulocyte-mediated cytotoxicity and augmented by trogocytosis [133,134] or immunological check point inhibition [135]. However, the pro-tumorigenic effects of PMN include enhancing local and intravascular invasion and survival, and seeding/metastasis of cancer cells mediated by an individual PMN subpopulation, PMN-MDSCs, and their released exosomes. We discuss in detail these issues in the following subsections.

#### 3.3.1. Molecular Mechanisms of Immunosuppression by PMN-MDSC Phenotype

MDSCs have been observed in blood, lymph nodes, bone marrow, and tumor tissues, in addition to placental decidual tissues that are generally identified by the co-expression of two cell surface-expressed markers, CD11b and Gr-1 [136]. The immunosuppressive activity of MDSCs is linked to the metabolism of L-arginine to L-ornithine to suppress the expression of T cell receptor ξ chain which in turn is necessary for T cell activation [137,138]. Besides, T cell functions could be impaired by these suppressive neutrophils via the interactions between T cell, PD-1 and PD-L1 on PMN-MDSCs for blocking T cell proliferation and cytokine production [139]. Bruno et al. [140] furthermore demonstrated that PMN-MDSC could interact with NK cells to impact the tumor development, angiogenesis and progression.

#### 3.3.2. Clinical Applications of Exosomes Released from PMN-MDSCs in Autoimmune and Inflammatory Diseases

The exosomes released from PMN-MDSCs (PMN-MDSC-Exo) are found immunosuppressive in nature. Wang et al. [141] by using dextran sulfate sodium (DSS)-induced murine colitis as experimental model found that PMN-MDSC-Exo-treated mice showed greater resistance to DSS-induced colitis. A decrease in the proportion of Th1 cells but an increase in regulatory T (Treg) cells was found in mesenteric lymph nodes of DSS colitis after PMN-MDSC-Exo administration. In addition, lower serum levels of IFN-γ and TNF-α were found related to the increased arginase-2 activity. The same authors then by using collagen-induced arthritis (CIA) as animal model of RA discovered that PMN-MDSC-Exo decreased the percentage of Th1 and Th17 cell populations in both in vivo and in vitro animal colitis model. This suppression is related to the content of miR-29a-3p and miR-93-5p in the released exosomes. The two exosome-derived microRNAs were confirmed to inhibit Th1 and Th17 cell differentiation by targeting T-bet and STAT3 [142]. These findings may suggest the therapeutic potential of PMN-MDSC derived exosomal microRNAs in treating immune-mediated inflammatory diseases. Zoller et al. [143] demonstrated that not only PMN-MDSC but also PMN-MDSC-Exo could become a potential therapeutic strategy for treating autoimmune alopecia areata in that autoreactive Th1 and type 1 CD8^+^ cytotoxic T cell (Tc1) expansion is a dominant feature in the sites of autoimmune-mediated hair follicles.

**Figure 9:**
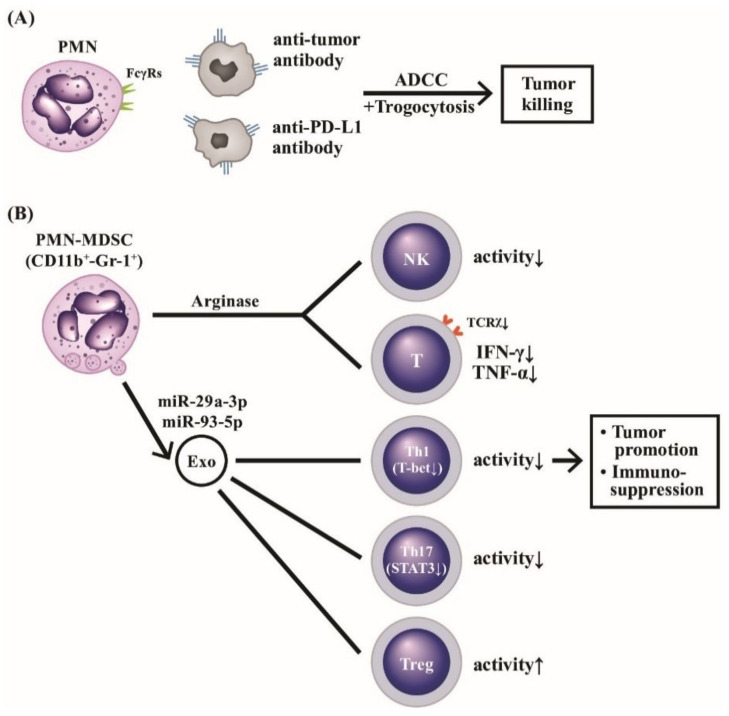
The molecular basis of PMNs on tumor killing, tumor promotion, and immunosuppression. PMNs can mediate tumor killing by way of anti-tumor antibody or anti-immunological checkpoint antibody (anti-PD-L1) via ADCC (**A**). Furthermore, the ADCC killing activity can augment polymorphonuclear myeloid-derived suppressor cells (PMN-MDSC with surface markers of CD11b^+^-Gr-1^+^), which release arginase and suppressive-type exosomes containing miR-29a-3p and miR-93-5p to potently suppress NK, T cells with TCR*x*, Th1 and Th17 subpopulations whereas Treg is conversely activated (**B**). These suppressive effects on immune-related cells facilitate immunosuppression and tumor promotion. TCR: T cell receptor; PD: programmed cell death protein; PD-L: programmed cell death protein receptor; NK: natural killer cell; IFN: interferon, TNF: tumor nerosis factor; miR: microRNA; Gr: granulocytic marker; STAT: signal transducer and activator of transcription; T-bet: T box expressed in T cell transcription factor; Treg: regulatory T cell.

The roles of PMN-MDSC and PMN-MDSC-Exo in cancer immunity, autoimmunity, and inflammatory diseases are depicted in Figure 9, unveiling the molecular basis of PMN and CD11b^+^-Gr-1^+^PMN-MDSC subset in tumor immunity. Normal PMNs can exhibit potent ADCC activity for tumor killing. The suppressive type PMN-MDSCs conversely promote tumor growth via immunosuppression.

## 4. The Cytotoxicity of PMN on the Other Particular Pathogens and/or Cells via Trogocytosis

*Trichomonas vaginalis* is a large unicellular motile eukaryotic parasite, which exclusively infects human reproductive system. Mercer et al. [144], by using 3D and 4D live imaging, observed that PMNs kill the parasites in a contact-dependent, NET-independent engulfment process with bites prior to parasite death, i.e., trogocytosis. In addition, Olivera-Valle et al. [145] watched that PMN “bites” sperm and quickly reduces its motility (<5 min) and viability (<20 min) after contact with it in the vaginal lumen but with a low impact on the mucosa damage. These observations indicate PMNs can act as vaginal patrolmen for keeping vaginal homeostasis.

## 5. The Roles of PMNs in Inflammation Resolution and Wound Healing

PMNs are traditionally regarded as potent effectors in the site of acute inflammatory reaction. However, several lines of evidence support that these acute inflammatory cells may also contribute to amelioration of inflammation and tissue repair after acute stage.

### 5.1. The Role of PMN in Inflammation Extinction in Acute Inflammatory Sites

It has been demonstrated that PMN’s effects on inflammation resolution are mainly by the release of PMN-derived pro-resolving product, annexin A1 [146], to promote PMN apoptosis, increase in the capacity of engulfment of apoptotic PMNs by macrophages, regulation of TNF-α and IL-6 production by monocytes, and down-regulation of mast cell degranulation. The next step involves the lipid class switching from classical prostaglandins and leukotrienes to pro-resolving lipoxins (Lx) mediated by 15-lipoxygenase (15-Lo) [147]. The generated lipoxin A4 (LxA4) further inhibits PMN recruitment into inflammation site [148]. Anti-inflammatory cytokine IL-10 production by PMN is also presumed to suppress inflammation [149]. In addition, the enhanced neutrophil apoptosis with engulfment by macrophages promotes TGF-β secretion for the abolishment of inflammation [150]. Cumpelik et al. [151] further demonstrated that PMN-derived ectosomes could resolve the acute gouty inflammation by inhibiting C5a-mediated inflammasome priming. Recently, Calvente et al. [152] revealed that hepatic PMNs would facilitate the proinflammatory macrophage into an anti-inflammatory phenotype via granulocytic microRNA-223.

The tissue damage caused by acute inflammation needs to be repaved once the stimulus itself has been removed. Although local macrophages are crucial in remodeling the tissue architecture for restoring functions, recent investigations revealed that PMNs are also important for tissue repair and regeneration.

### 5.2. The Role of PMN on Wound Healing

**Figure 10:**
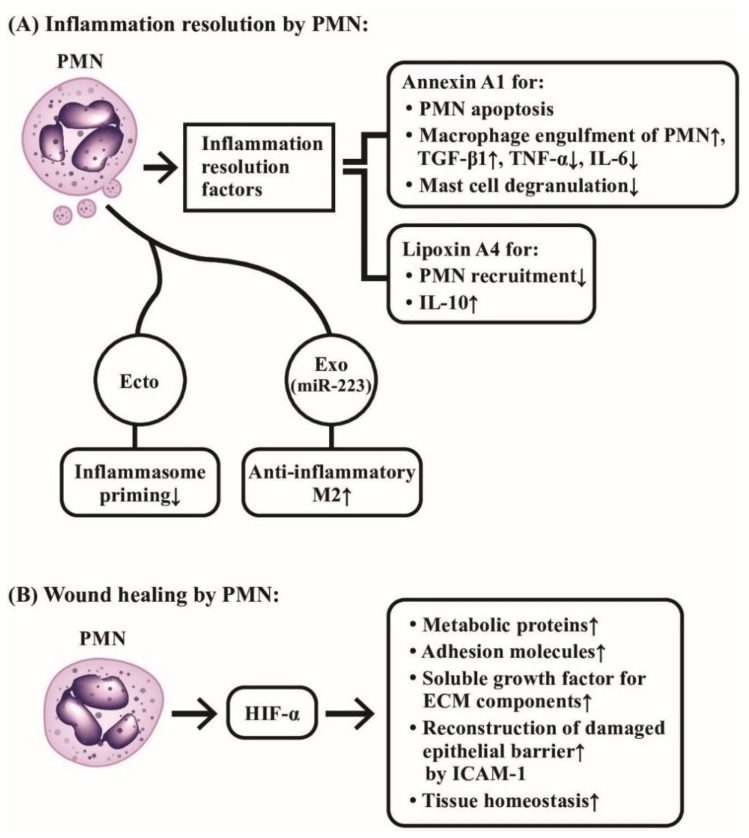
The molecular mechanism of neutrophils in inflammation resolution and wound healing. (**A**) Neutrophils release annexin A1 and lipoxin A4 as inflammation-resolution factors to suppress the inflammatory functions of PMNs, macrophages and mast cells. In addition, PMN-released ectosomes (Ecto), and exosomes (Exo) containing miR-223 also play important roles in suppressing inflammation. (**B**) Neutrophil-released hypoxia-induced factor-α (HIF-α) mediates a crucial role in tissue homeostasis and wound healing through induction of many tissue repair molecules after inflammation resolution. ECM: extracellular matrix; ICAM: intercellular adhesion molecule; miR: microRNA; M2: type 2 macrophage.

Horckmans et al. [153] demonstrated that PMNs could promote myocardial healing after ischemic infarct in mice. Botusan et al. [154] and Hong et al. [155] discovered that hypoxia-induced factor 1α (HIF-1α) was a critical factor for wound healing in diabetes mouse since HIF-1α could up-regulate metabolic proteins, adhesion molecules, soluble growth factors, and extracellular matrix (ECM) components. Therefore, PMN-mediated HIF-1α stabilization in wound micro- environments acts through epithelial cells for promoting damaged barrier restitution and tissue homeostasis. Sumagin et al. [156] demonstrated that epithelial intercellular adhesion molecule-1 (ICAM-1) played an important role in activating epithelial Akt and β-catenin signaling pathway to mediate epithelial proliferation and wound repair. The involvement of PMNs in the inflammation resolution and wound healing are demonstrated in Figure 10, which highlights the molecular basis of the two novel biological activities of PMNs on anti-inflammation and wound healing instead of eliciting inflammatory reaction.

## 6. The Roles of Neutrophil-Platelet Interactions in Hemotasis, Vascular Inflammation, and Atherogenesis

Platelets are non-nucleated fragment in plasma derived from bone marrow megakaryocytes. Upon activation, platelets can express many adhesion molecules, including P-selectin (CD11b), β2 (CD18/CD41), and β3 integrins (CD61) that can interact with neutrophils [157]. Once platelets adhere to P-selectin glycoprotein ligand-1 (PSGL-1) on PMNs, PMNs activation is induced by enhancing tyrosine phosphorylation and MAP kinase in the sites of acute inflammation to prevent bacterial invasion [158]. On the other hand, the activated platelets may be involved in hemostasis, wound healing, and inflammation [159].

### 6.1. Dysregulated Neutrophil-Platelet Interactions Foster Sterile Inflammation and Tissue Damage in Immune-Mediated Vascular Diseases

Totani et al. [160] demonstrated that aberrant neutrophil-platelet interactions are a hallmark of cardiovascular diseases. Maugeri et al. [161] discovered that both leukocytes and platelets were activated in patients with giant cell arteritis and polymyalgia rheumatica. In addition, persistent systemic inflammation in different autoimmune diseases such as SLE [162,163], systemic sclerosis [164,165], and RA [166,167] are all related to dysregulated neutrophil-platelet interactions in fostering sterile inflammation and tissue damage.

### 6.2. Role of Neutrophils in Atherogenesis

It is conceivable that activated neutrophils produce and release reactive oxygen species (ROS), inflammatory leukotrienes, and proteolytic lysosomal enzymes, which can directly damage vascular endothelial cells. As discussed in the previous subsections, the heterotypic interaction between PMNs and platelets might represent a link between hemostasis/thrombosis and inflammatory reactions. Kostis et al. [168] revealed that the association of leukocyte count and the extension of coronary atherosclerosis could be observed by coronary arteriography. Naruko et al. [169] even found neutrophil infiltration in the atherosclerotic lesions in acute coronary syndromes. van Leeuwen et al. [170] observed the accumulation of MPO-positive neutrophils in atherosclerotic lesions in low-density lipoprotein receptor −/− (LDLR−/−) mice. Carbone et al. [171] found that PMNs played a crucial role in accelerating plaque vulnerability via release of different enzymes such as gelatinase, collagenase, elastase, and myeloperoxidase in both early and late stage of atherogenesis to destabilize the mature plaques. Marino et al. [172] further demonstrated that the circulating and intra-plaque neutrophils in patients with carotid atherosclerosis produced IL-8, VEGF and elastase crucial for plaque development and progression. Mittal et al. [173] discovered that transient receptor potential melastatin-2 (TRPM2) expressed in endothelial cells can induce neutrophil activation, trans-endothelial migration, and vascular injury. These observations provide a prospective therapeutic potential in the future for blocking TRPM2 activation in endothelial cells to modify PMN-mediated vascular damage.

**Figure 11:**
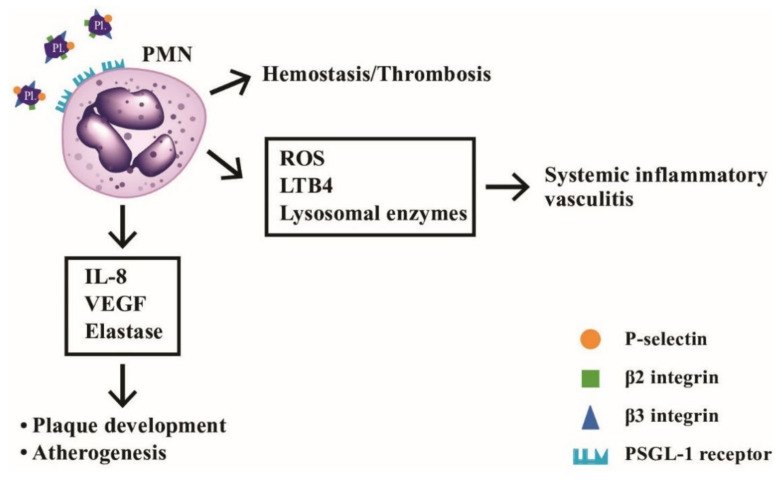
The interactions between neutrophils and platelets in hemostasis, thrombosis, systemic inflammatory vasculitis, and atherogenesis via binding between adhesion molecules (P-selectin, β2-integrin and β3-integrin) on platelets and P-selectin glycoprotein ligand-1 (PSGL-1) on neutrophils. After ligand-receptor binding, the released mediators including ROS, LTB4, and lysosomal enzymes can induce inflammatory vasculitis, whereas other factors including IL-8, VEGF and elastase can facilitate atheroma plaque development and atherogenesis in the blood vessels. LTB4: leukotriene B4; VEGF: vascular endothelial growth factor; ROS: reactive oxygen species; PL: platelet.

The platelet-neutrophil interactions involved in the hemostasis, vascular inflammation, and atherogenesis is shown in Figure 11. It depicts the molecular bases of interaction between PMNs and platelets underlying hemostasis, systemic vasculitis, and artherogenesis.

## 7. Conclusions and Prospects

PMNs have long been regarded as the first-line defending phagocytic cells against bacterial pathogens. Recently, these traditional acute inflammatory cells have been found to be heterogeneous and able to cross-talk with different immune-related cells, somatic cells, and tumor cells to mediate immune responses, inflammation/anti-inflammation, tumor immunity, hemostasis, tissue homeostasis/wound healing, autoimmunity, inflammatory diseases, and cardiovascular diseases. In addition to releasing cytokines, chemokines, and growth factors to participate in an immune network, PMNs also undergo exocytosis to secrete microvesicles, including exosomes and ectosomes, which are also able to affect the physiology/pathophysiology of remote cells. It will be quite valuable to elucidate further the biological/pathobiological roles of the heterogeneous neutrophil subpopulations including LDG, MDSC, and other cells. In addition, the identification and verification of these neutrophil subpopulations as disease biomarkers in the atherosclerotic plaque will also be a future strategy for early detection and preclusion of deterioration for cardiovascular diseases.

## Abbreviations

AAV	anti-neutrophil cytoplasmic antibody associated vasculitis
ADCC	antibody-dependent cell-mediated cytotoxicity
AOSD	adult onset Still’s disease
APC	antigen presenting cell
BLyS	B lymphocyte stimulator
CSF	colony stimulating factor
CVD	cardiovascular disease
DAMP	damage-associated molecular pattern
DC	dendritic cell
DTH	delayed-type hypersensitivity
ECM	extracellular matrix
Ect	ectosome
Exo	exosome
FcγR	IgG Fc receptor
G-CSF	granulocyte colony stimulating factor
GM-CSF	granulocyte monocyte colony stimulating factor
HDG	high density granulocyte
HLA	human leukocyte antigen
IBD	inflammatory bowel disease
IEC	intestinal epithelial cell
IFN	interferon
IL	interleukin
IL-1ra	interleukin 1 receptor antagonist
LDG	low density granulocyte
LF	lactoferrin
LFA	leukocyte functional antigen
LPS	lipopolysaccharide
LTB4	leukotriene B4
Lx	lypoxin
M-CSF	monovcyte cvolony stimulating factor
MMP	matrix metalloproteinase
MNC	mononuclear cell
MPO	myeloperoxidase
NET	neutrophil extracellular trap
NK	natural killer
Nox	NADPH oxidase
PMN	polymorphonuclear neutrophil
PMN-MDSC	polynorphonuclear myeloid-derived suppressor cell
PSGL-1	P-selectin glycoprotein ligand-1
RA	rheumatoid arthritis
RANKL	receptor activator of nuclear factor kappa B ligand
ROS	reactive oxygen species
SLE	systemic lupus erythematosus
Tc	cytotoxic T cell
TGF-β1	transforming growth factor beta 1
TK	tyrosine kinase
TLR	Toll-like receptor
TNF-α	tumor necrosis factor alpha
Treg	regulatory T lymphocyte
VEGF	vascular endothelial growth factor
